# Pediatric eosinophilic esophagitis outcomes vary with co-morbid eczema and pollen food syndrome

**DOI:** 10.3389/falgy.2022.981961

**Published:** 2022-09-02

**Authors:** Julia Sessions, Natasha Purington, Yiwen Wang, Sean McGhee, Sayantani Sindher, Alka Goyal, Nasim Khavari

**Affiliations:** ^1^ Stanford School of Medicine, Stanford Medicine, Stanford, CA, United States; ^2^ Department of Pediatrics, University of Virginia, Charlottesville, VA, United States; ^3^ Stanford Quantitative Sciences Unit, Stanford University School of Medicine, Stanford, CA, United States; ^4^ Division of Allergy, Immunology, & Rheumatology, Stanford Medicine, Stanford, CA, United States; ^5^ Pediatric Gastroenterology, Hepatology, & Nutrition, Stanford Medicine, Stanford, CA, United States

**Keywords:** eosinophilic esophagitis (EoE), eczema, oral allergy syndrome (OAS), outcomes, atopic dermatitis, allergy, pollen food syndrome (PFS), pollen food allergy syndrome (PFAS)

## Abstract

**Background:**

Eosinophilic esophagitis (EoE) is a chronic immune-mediated inflammatory disease characterized by eosinophil inflammation of the esophagus. It has been described as a component of the Allergic March and is often seen with other atopic diseases. Some atopic diseases, including asthma, are known to be heterogenous with endotypes that guide treatment. Similarly, we propose that EoE is a heterogenous disease with varying phenotypes and endotypes that might impact response to therapy.

**Methods:**

A single-center retrospective review of pediatric patients ≤18 years of age diagnosed with EoE was conducted. All gastrointestinal clinic visits and esophagogastroduodenoscopies (EGD) from disease presentation through the first three years after diagnosis were reviewed. Histologic remission rate and therapies utilized [proton pump inhibitor (PPI), topical steroid, dietary elimination] were assessed.

**Results:**

One hundred and thirty-seven patients were included, 80% of whom had at least one concurrent atopic condition at diagnosis, with food allergies being the most common (57%) followed by eczema (34%), and asthma (29%). The remission rate of the overall cohort was 65%, and by concurrent allergy, comorbid pollen food syndrome and eczema had the highest remission rates at 100% and 81%, respectively followed by asthma (62%), food allergies (62%), seasonal allergic rhinitis (60%), and history of anaphylaxis (56%). Kaplan-Meier curves for each atopic condition show that patients with eczema and pollen food syndrome achieve histologic remission faster than those without. All treatment modalities were more successful in patients with eczema than those without, and PPI was most effective treatment at inducing remission.

**Conclusions:**

In a real-world pediatric cohort, 80% of patients with EoE had an underlying atopic condition. Patients with eczema and pollen food syndrome had a swifter response and were more likely to achieve histologic remission than patients with other atopic conditions. This study suggests that EoE, like other allergic diseases, may have heterogenous phenotypes that could affect response to treatment. There is currently a knowledge gap in classifying EoE based on endotypes and phenotypes at diagnosis and correlating responses to various treatment modalities.

## Introduction

Eosinophilic esophagitis (EoE) is a chronic immune mediated inflammatory disease of esophageal eosinophil infiltration (1). It has been described as a component of the Allergic March and is often known to affect patients with underlying atopic comorbidities (2, 3). EoE is defined by clinical symptoms of esophageal dysfunction and histologically by presence of ≥15 eosinophils (eos)/high-powered-field (hpf) in the esophageal biopsies (4, 5). Recent literature has described heterogeneity in EoE patients, with variations in disease phenotypes and endotypes (6). Disease variations are well described in other atopic diseases, with endotypes and inflammatory markers that guide clinical treatment (7). For example, asthma endotypes of Th2 high (eosinophilic) vs. Th2 low (non-eosinophilic) are well established and there are now biologic agents used for patients with Th2 high disease (8). Likewise, EoE may be classified based on phenotypic (age, severity, response to therapy, fibrosis, and/or atopic comorbidities) or pathogenic variability resulting in endotypes (Th2 high vs. low, abnormal epithelial barrier function, esophageal fibrosis, genetic markers) (6). This study aims to investigate whether differences in type of underlying allergic manifestations could be correlated to disease endotype, course, and response to therapy.

## Materials and methods

### Patient population and data sources

Stanford University Institutional Review Board for Human Subjects Research provided ethical approval for this retrospective review (IRB 61 Registration #4947). The Stanford database identified patients ≤18 who had an esophagogastroduodenoscopy (EGD) and EoE diagnosis by ICD9 or ICD10 code. Patients were included if their diagnostic EGD had ≥15 eos/hpf in at least one esophageal biopsy sample along with the presence of esophageal symptoms, based on the current diagnostic criteria for EoE (4, 5, 9). Exclusion criteria include patients with complex systemic diseases such as inflammatory bowel disease, celiac disease or history of solid organ transplantation, as well as patients diagnosed with gastroesophageal reflux disease rather than EoE based on experienced clinician review (10–12).

Chart review involved data collection to investigate demographics, anthropometrics, symptoms, current medications, past medical history, and relevant family history for the visit prior to the diagnostic EGD, as well as for all gastrointestinal clinic visits and endoscopies for up to three years post-diagnosis. Stanford has a pediatric EoE clinic, which is a multidisciplinary clinic with a gastroenterologist and allergist, and the majority of clinic visits reviewed were a part of the combined clinic. Classification of co-morbid allergy at diagnosis was based on history of atopic conditions in the initial clinic note, which was either parent reported or based on physician chart review. Endoscopic data collected and reviewed included operative and histopathology reports with peak eos/hpf (13). Macroscopic inflammation seen endoscopically was described as edema, rings, exudate, furrows, and stricture, all of which were included in the data collected (14–16).

### Treatment modality

Treatment information was collected from endoscopy or clinical visit records and treatment duration was calculated based on visit dates and notes directly. It was standard of care for the provider to inquire about therapy adherence on a yes/no basis at each visit, and only patients who stated they were adherent to the prescribed therapy were included in analysis for that treatment. Only treatments tried for at least six weeks prior to either the date of first histological remission or date of last visit record were considered as full treatment. If a treatment was tried for less than six weeks, it was labeled as partial treatment, and if there was only one record of a treatment, it was labeled as unknown treatment duration. If a patient was eliminating specific foods at the initial visit, these foods were not considered as part of an elimination diet. Treatment options analyzed include topical steroids (TS) (swallowed inhalational fluticasone or budesonide slurry), dietary elimination (DE), proton pump inhibitor (PPI), and combination treatment. Visit records were sorted by date for each patient, and the cumulative time that the treatment was tried was calculated for each of the three main treatment modalities across visits (PPI, DE, and TS). Combination therapy was assessed by determining the amount of time multiple therapies were tried concurrently for a minimum of six weeks. Remission was defined as <15 eos/hpf on repeat endoscopic evaluation. PPI responsiveness was defined as at least 6 weeks of treatment with a PPI in mono- or combination therapy that resulted in histologic remission.

### Statistical methods

Baseline characteristics were summarized for the overall cohort using counts and percentages or medians and inner quartile ranges (IQR), as applicable. The percentage of patients in histologic remission for each atopic condition reported at EoE presentation was descriptively summarized. Similar methods were used to describe remission rates by eczema status and treatment group.

Time to first histologic remission was defined as the number of months from EoE presentation until the first visit in which peak eosinophil count was <15 eos/hpf. Patients who did not achieve histologic remission in the observation period were censored at the last EGD visit recorded within the first three years after diagnosis. Time to first remission was compared among different atopic conditions using Kaplan-Meier survival estimates which included: anaphylaxis, asthma, eczema, food allergy, pollen food syndrome (PFS, also known as oral allergy syndrome), and seasonal allergic rhinitis. The log-rank test was used to determine whether time until remission differed by the presence or absence of each of the six atopic conditions. Median months until remission and 95% confidence intervals (CIs) were reported. If significant differences in remission rates were found, each atopic condition was further analyzed to correlate whether rate and timing of response with the treatment modality (PPI, DE, or TS). Patients with incomplete records on response status or treatment utilized were excluded from the treatment analyses. If the log-rank test for atopic condition and treatment was statistically significant, post-hoc pairwise tests were conducted and *p*-values were adjusted for multiple testing using the Benjamini-Hochberg method for controlling the false discovery rate (17).

A *p*-value <0.05 was considered statistically significant. All analyses were conducted in R v4.0.2 (18).

## Results

### Patient characteristics and allergic co-morbidities

**Figure 1:**
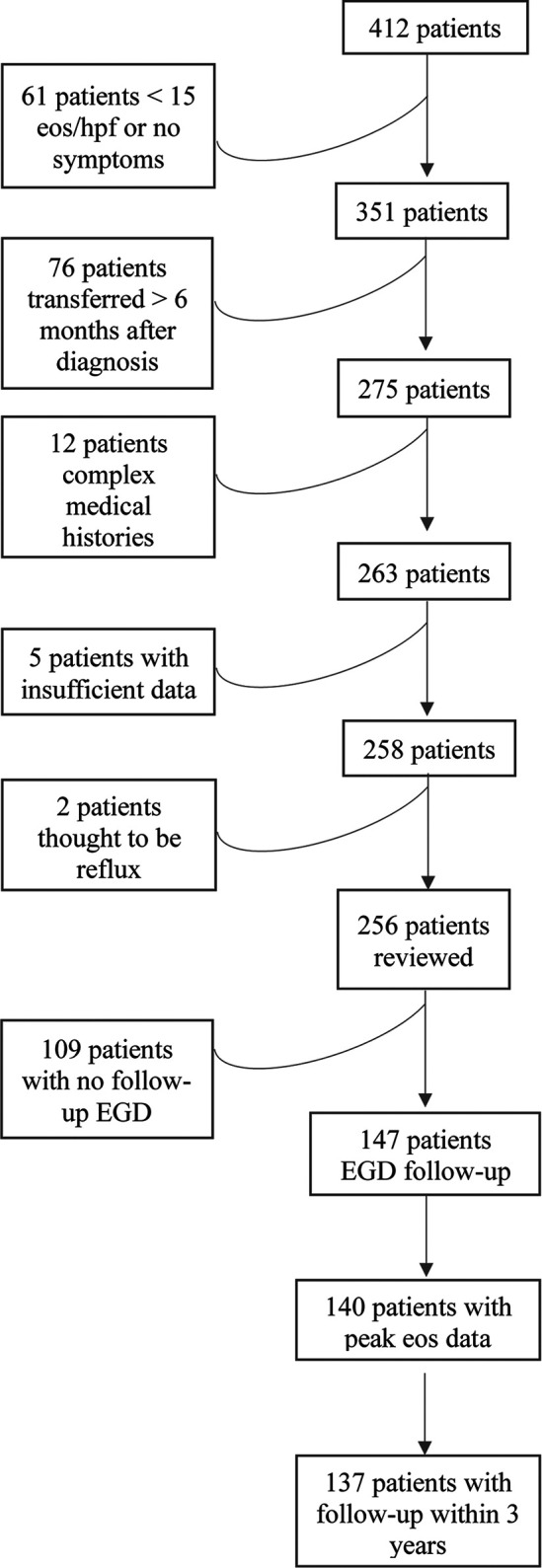
Flow diagram of patient inclusion. Flow chart indicating why patients were excluded from the retrospective review. For the two patients excluded by clinician review, the peak eosinophils/hpf were 15 and 16, respectively, and the patients had mild symptoms that improved with anti-reflux treatment.

The Stanford database identified 412 pediatric patients with an ICD9 or ICD10 code for EoE who had undergone an EGD, 256/412 fulfilled histological criteria for EoE diagnosis and were included in the review, which took placed between June 2018 and October 2019. Of these, 147/256 had repeat EGDs, and 137/147 had sufficient follow-up and/or EGD data (Figure 1). Within the total 256-patient cohort, 43/256 (17%) of patients were diagnosed within 1 year of chart review and 43/256 (47%) within 3 years. Within the 137-patient cohort with adequate follow-up data, the average age of diagnosis was 6.4 years, most of the patients were male (74.5%) and Caucasian (33%) or an unknown race (35%) (Table 1). Concurrent allergies were prevalent, with up to 80% of patients having a comorbid allergy diagnosed. The most common atopies were food allergy (57%), eczema (34%), asthma (29%), and seasonal allergic rhinitis (24%). Specific counts of each combination of comorbid atopic diagnosis are present in Supplementary Figure S1.

**Table 1:** Baseline characteristics.

Baseline characteristics	Overall (*n* = 137)
Age at diagnosis (years), Median [Min, Max]	6.4 [0.7, 17.0]
Male	102 (74.5%)
Race/ethnicity	
Asian	16 (11.7%)
Black/African	3 (2.2%)
Caucasian	45 (32.8%)
Hispanic/Latino	8 (5.8%)
Multiple	17 (12.4%)
Unknown	48 (35.0%)
BMI (>2 yo) or weight-for-length (<2 yo) percentiles	
<1	7 (5.1%)
1–10	25 (18.2%)
10–25	16 (11.7%)
25–75	38 (27.7%)
75–90	12 (8.8%)
90+	11 (8.0%)
Atopic symptoms	109 (79.6%)
Anaphylaxis	18 (13.1%)
Asthma	40 (29.2%)
Eczema	47 (34.3%)
Seasonal allergic rhinitis	33 (24.1%)
Food allergies	78 (56.9%)
Pollen food syndrome	7 (5.1%)
Unknown allergies	5 (3.6%)
Diagnosis of, *n* (%)	
Celiac disease	1 (0.7%)
EGID (eosinophilic gastro-intestinal disorder)	9 (6.6%)
Esophageal malformation (esophageal atresia or trachea-esophageal fistula), *n* (%)	6 (4.4%)
Family history of, *n* (%)	
EoE	6 (4.4%)
Atopic condition	56 (40.9%)
Peak eosinophil count on endoscopy, median [range]	48 [14, 216]
Acute presentation in ED/IP admission, *n* (%)	15 (10.9%)
EGD Gross Findings	
Stricture/Narrowing	8 (5.8%)
Rings/trachealization	18 (13.1%)
Linear furrow	66 (48.2%)
Mucosal fragility	14 (10.2%)
Exudate/Microabscess	38 (27.7%)
Food impaction	6 (4.4%)
Erythema	10 (7.3%)
Edema	13 (9.5%)
Total number of pediatric GI endoscopies, median (range)	3 [1, 7]

### Histologic remission

Eighty nine of 137 patients (65%) went into remission at some point during the observation period. Supplementary Figure S2 shows rates of histologic remission at any point in the observation period based on presence or absence of comorbid atopic diagnoses. Histologic remission was achieved in 100% (7 of 7) in patients with pollen food syndrome (PFS) vs. 63% of those without PFS and 81% in patients with eczema vs. 56% of those without eczema (Supplementary Figure S2). Rates of histologic remission in patients with and without anaphylaxis, asthma, food allergies, and seasonal allergic rhinitis were similar. There was not an association between number of allergic diagnoses (ranging from zero to six), remission rate, or time to remission (Supplementary Table S1). Symptom duration by comorbid atopic diagnosis was similar for all allergies, with most patients (42–45%) having symptoms for >12 months prior to diagnosis.

**Figure 2:**
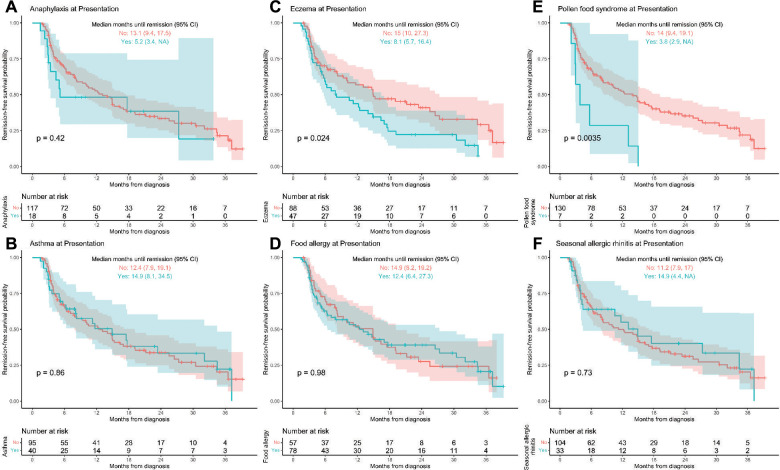
(**A–F**) Time to first remission by atopic/allergic diagnoses at presentation. Kaplan-Meier curves of time until first histologic remission by the six main allergic/atopic diagnoses at presentation with 95% confidence bands. Children that did not experience histologic remission by 3 years post-diagnosis were censored at their last available EGD visit. “NA” indicated that the upper confidence limit is undefined.

Correlation with comorbid atopy at the time of presentation, showed significantly faster time to first remission in patients with eczema (*p* = 0.024) and PFS (*p* = 0.0035) (Figures 2C,E). Patients who reported eczema at presentation went into remission a median of 7 months faster than those who did not report eczema (8.1 [95% CI, 5.7–16.4] vs. 15 [10.0–27.3] months, respectively; *p* = 0.024). Median time to first remission was 3.8 (2.9, upper limit undefined) months in PFS patients compared to 14 (9.4, 19.1) months in non-PFS (*p* = 0.0035). Demographics for eczema vs. non-eczema and PFS vs. non-PFS patients is seen in Supplementary Table S2 and shows eczema patients had a younger average age of diagnosis at 4.6 years compared to 7.1 years for non-eczema patients (*p* = 0.004) and PFS patients had lower peak eos/hpf on initial endoscopy compared to non-PFS patients (*p* = 0.002) (Supplementary Table S2). Patients with eczema had trends towards higher peak eosinophil count on initial endoscopy compared to non-eczema patients (55 eos/hpf vs. 45 eos/hpf, *p* = 0.39) and less frequent or similar EGD macroscopic findings in all fields (stricture/narrowing, rings/trachealization, linear furrow, mucosal fragility, exudate/microabscess, food impaction, edema, erythema) (Supplementary Table S2). The outcomes for patients with eczema appeared promising while no conclusions could be drawn due to a small sample size in patients with PFS. PFS comorbid atopic conditions and treatment that induced remission is available in Supplementary Table S3.

### Eczema and treatment

**Figure 3:**
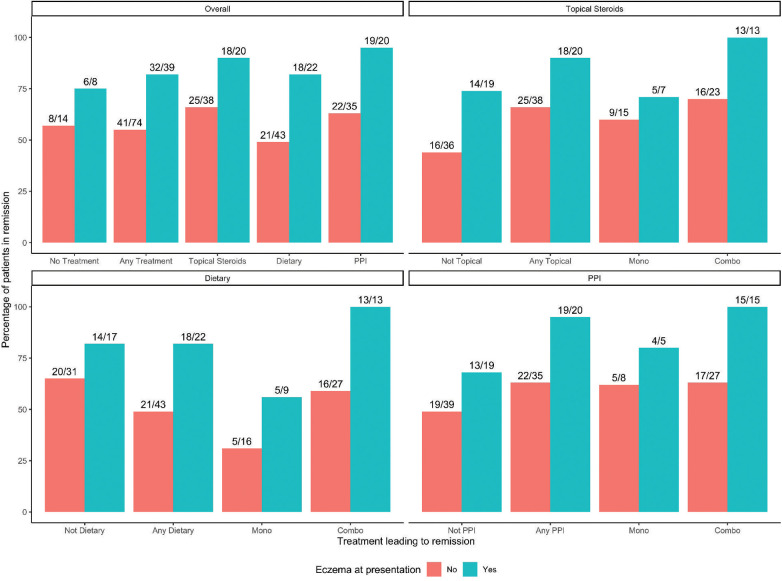
Eczema status and remission rate by treatment group. Bar graphs of history of eczema at presentation and therapies by remission rate. Overall includes any therapy and no treatment.

**Figure 4:**
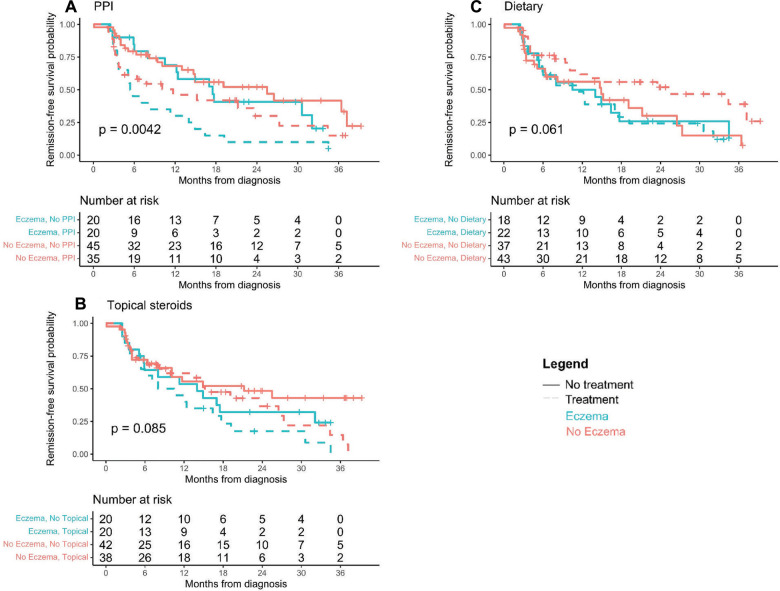
(**A–C**) Time to first remission by last full treatment tried. Kaplan-Meier curves of time until first histologic remission by treatment type and eczema status. Children that did not experience histologic remission by 3 years post-diagnosis were censored at their last available EGD visit.

Investigations into patients with eczema and specific treatments that led to histologic remission show that all therapies are successful in patients with eczema compared to those without eczema. No patients were on biologic immunomodulator medications as a first-line therapy. Figure 3 shows that overall (any treatment), TS, DE, and PPI each in monotherapy or in combination therapy had higher remission rates in patients with eczema than those without. One hundred percent of eczema patients on combination therapy achieved histologic remission (Figure 3). Kaplan-Meier curves of time to first histologic remission by treatment type (mono or combination therapy) and eczema status are shown in Figure 4. There were significant differences in time to first remission by eczema status and use of PPI (Figure 4A). Among patients with eczema, those on PPI had the shortest median time to remission [5.6 months (3.7–14.9)] compared to non-eczema patients not on PPI [25.5 months (14.7-undefined)] (post-hoc *p*-value = 0.0019). There was no significant difference among eczema patients treated vs. not treated with PPI (post-hoc *p*-value = 0.056) or among eczema vs. non-eczema patients on PPI therapy (post-hoc *p*-value = 0.22). Looking at the overall cohort, most of the patients treated with PPI did go into remission, with 41/55 (74.5%) treated with PPI in mono- or combination therapy achieving histologic remission; demographics and baseline data for patients who were PPI responsive vs. non-responsive is shown in Supplementary Table S4, with 46% of PPI responsive patients having eczema compared to 7% of PPI non-responsive.

## Discussion

EoE is a relatively new chronic inflammatory disease and like other allergic conditions it may have underlying heterogeneous phenotypes, endotypes and outcomes. This study shows significantly higher and faster histologic remission rates in patients with underlying eczema and PFS. The differential response to treatment may suggest a different underlying endotype of EoE in the setting of eczema.

Studies have investigated atopic vs. non-atopic sub-populations of EoE patients. Ancellin et al. showed 78% of a pediatric EoE cohort had concurrent atopic diseases, those with atopic disorders had non-significant higher remission rate that those without, and PPI was commonly used as a first-line therapy (19). Notably, Ancellin et al. grouped all atopic disorders together whereas this study further investigates different atopic disorders and shows significantly higher and faster histologic remission rates in patients with history of eczema and PFS (19).

Eczema is an atopic disease specifically related to epidermal barrier function and, in our study, EoE patients with eczema are younger, have fewer macroscopic findings on initial endoscopy, and are more treatment responsive with favorable outcomes. Typical therapy for both eczema and EoE includes medications, such as steroids and dupilumab (a monoclonal antibody against IL4 and IL13), known to also be effective in Th2-driven atopic inflammation (20, 21). Our results suggest that the presence of eczema may identify those patients who have primarily atopic inflammatory EoE. A possible mechanistic link between eczema and EoE responsiveness lies in tissue barrier function *via* filaggrin, an epithelial protein that aids in the structure and function of the stratum corneum and plays a key role in pathogenesis of eczema and food allergy related inflammation (22, 23). Th2 inflammation down-regulates filaggrin, which leads to barrier defects in eczema. Eczema treatment is directed towards decreasing inflammation and restoring the skin barrier, which allows for restoration of filaggrin function (22). A similar barrier defect may be driving inflammation in EoE and the profilaggrin gene *FLG* has been linked to EoE patients (24). Similar to eczema, in EoE as therapies decrease inflammation, filaggrin function may improve and the barrier defect restored. Thus, the mode of therapy (PPI, TS, DE) may be less important as long as inflammation decreases. Our preliminary data is promising in that atopic EoE patients may have different phenotypes, and future studies would be helpful to develop more patient and symptom specific guidelines. For example, patients without eczema may require other therapies or more careful monitoring to ensure their response.

There were few patients with PFS in this cohort, reducing our ability to draw general conclusions about these patients. Other studies have noted a strong association between PFS and EoE, including higher rates of PFS in EoE patients. Letner et al. looked at 346 adults with EoE and found 26% had concurrent PFS and EoE patients with PFS had higher rates of diagnosis in the Spring (25). Mahdavinia et al. looked at 186 adult EoE patients compared to adults with allergic rhinitis and found that PFS among pollen-sensitized cases was significantly more prevalent in the EoE (51%) compared to allergic rhinitis (10%) group (26). They propose that EoE may start with sensitization to pollen aeroallergens that cross-react with food proteins leading to esophageal inflammation/EoE before the food proteins are degraded in the stomach; this progression takes time so their group suggests that high rates of PFS and EoE may be seen in adult rather than pediatric EoE patients (26). Another possibility is that epithelial barrier disruption seen in EoE allows for sensitization to food proteins (26). Additional work is needed that might look at pediatric EoE populations and the role played by cross-reacting antigens, such as profilins, known to be fundamental in PFS. The low frequency of PFS in our cohort may have been due to data collection as only those with very prominent PFS had their disease noted in the routine medical record. Nonetheless, the very strong correlation between PFS and EoE remission in this cohort argues for more attention to the role these antigens may play in EoE.

In addition to favorable overall outcomes in EoE patients with eczema, this study also shows positive treatment response to PPI. EoE patients with eczema treated with PPI had higher rates of histologic remission than non-eczema patients who were not treated with PPI. There has been significant research into PPI-responsive EoE (PPI-REoE). In 2018 the EoE international diagnosis criteria changed to exclude a PPI trial prior to diagnosis of EoE, allowing for PPI to be accepted as treatment for EoE and a new group of PPI-REoE patients to exist (5). Literature shows that 23%–68% of pediatric EoE patients respond to PPI therapy with difficulty elucidating what predicts responders vs. non-responders (27). This data shows a high PPI response rate, with up to 74.5% of those treated with PPI in mono- or combination therapy achieving histologic remission and similar demographics between PPI-REoE and non-responsive (Supplementary Table S4). Despite the similarity in demographics, there were higher rates of eczema in the PPI-REoE group compared to the PPI non-responsive group. In this study, the majority of pediatric EoE patients respond positively to treatment with PPI, either in mono- or combination therapy, suggesting that PPI may be a useful component of therapy in EoE patients.

Overall, this data shows that there is heterogeneity in endotype in EoE patients, and that this variation may be related to the presence of Th2 type inflammation. EoE patients with eczema are significantly more treatment responsive with positive disease outcomes. The heterogeneity in EoE outcomes suggests a possible endotype variation that could be further studied with specific investigations into atopic biomarkers. Limitations of this study include the retrospective review format with missing data in medical records contributing to our low patient counts in treatment groups and variability in timing of endoscopic follow-up and treatments. Given the low number of patients and practice variation in treatment, patients were usually not on monotherapy, but various types of combination therapy were often utilized. Although allergy history was patient reported, many patients were seen in multidisciplinary EoE clinic with both a gastroenterologist and allergist who, together, reviewed the history. Furthermore, AGREE guidelines were instituted during the time of this study, meaning there may be inaccurate numbers of PPI responsive EoE patients included given that some patients were pre-treated with PPI before diagnosis (5). These factors hindered the ability to draw conclusions on correlations between phenotype, endotype and specific treatment response. Additionally, 43% of patients were lost to endoscopic follow-up, which further decreased our cohort size in assessment of histologic remission. Although there were many patients without endoscopic follow-up data, this may have been because 17% of the cohort was diagnosed within one year of chart review and 47% within 3 years; these patients may not have had sufficient time to present for follow-up. Furthermore, in comparison with current literature, Votto et. al had similar follow-up rates with just 40% of their 52-patient cohort having repeat endoscopic evaluation within one year of diagnosis (28). However, this study remains the first real-world study looking at treatment response based on different allergic phenotypes and to observe that patients with eczema and PFS appear to have a better outcome with some advantage to including PPI in the treatment of these patients.

## Data Availability

The raw data supporting the conclusions of this article will be made available by the authors, without undue reservation.

## References

[1] Leigh LY Spergel JM. An in-depth characterization of a large cohort of adult patients with eosinophilic esophagitis. Ann Allergy Asthma Immunol. (2019) 122(1):65–72.e1. 10.1016/j.anai.2018.09.452 30223114

[2] Hill DA Grundmeier RW Ramos M Spergel JM. Eosinophilic esophagitis is a late manifestation of the allergic march. J Allergy Clin Immunol Pract. (2018) 6(5):1528–33. 10.1016/j.jaip.2018.05.010 29954692 PMC6131029

[3] Capucilli P Cianferoni A Grundmeier RW Spergel JM. Comparison of comorbid diagnoses in children with and without eosinophilic esophagitis in a large population. Ann Allergy Asthma Immunol. (2018) 121(6):711–6. 10.1016/j.anai.2018.08.022 30194971

[4] Liacouras CA Furuta GT Hirano I Atkins D Attwood SE Bonis PA Eosinophilic esophagitis: updated consensus recommendations for children and adults. J Allergy Clin Immunol. (2011) 128(1):3–20.e6. 10.1016/j.jaci.2011.02.040 21477849

[5] Dellon ES Liacouras CA Molina-Infante J Furuta GT Spergel JM Zevit N Updated international consensus diagnostic criteria for eosinophilic esophagitis: proceedings of the AGREE conference. Gastroenterology. (2018) 155(4):1022–33.e10. 10.1053/j.gastro.2018.07.009 30009819 PMC6174113

[6] Ruffner MA Cianferoni A. Phenotypes and endotypes in eosinophilic esophagitis. Ann Allergy Asthma Immunol. (2020) 124(3):233–9. 10.1016/j.anai.2019.12.011 31862435

[7] Woodruff PG Modrek B Choy DF Jia G Abbas AR Ellwanger A T-helper type 2-driven inflammation defines major subphenotypes of asthma. Am J Respir Crit Care Med. (2009) 180(5):388–95. 10.1164/rccm.200903-0392OC 19483109 PMC2742757

[8] Kuruvilla ME Lee FE-H Lee GB. Understanding asthma phenotypes, endotypes, and mechanisms of disease. Clin Rev Allergy Immunol. (2019) 56(2):219–33. 10.1007/s12016-018-8712-1. ``Understanding 30206782 PMC6411459

[9] Kim HP Dellon ES. An evolving approach to the diagnosis of eosinophilic esophagitis. Gastroenterol Hepatol. (2018) 14(6):358–66. PMID: 30166949 PMC6111507

[10] Dellon ES Katzka DA Collins MH Hamdani M Gupta SK Hirano I, on behalf of the MP-101-06 Investigators. Budesonide oral suspension improves symptomatic, endoscopic, and histologic parameters compared with placebo in patients with eosinophilic esophagitis. Gastroenterology. (2017) 152(4):776–786.e5. 10.1053/j.gastro.2016.11.021 27889574

[11] Wong J Goodine S Samela K Vance KS Chatfield B Wang Z Efficacy of dairy free diet and 6-food elimination diet as initial therapy for pediatric eosinophilic esophagitis: a retrospective single-center study. Pediatr Gastroenterol Hepatol Nutr. (2020) 23(1):79–88. 10.5223/pghn.2020.23.1.79 31988878 PMC6966220

[12] Gonsalves NP Aceves SS. Diagnosis and treatment of eosinophilic esophagitis. J Allergy Clin Immunol. (2020) 145(1):1–7. 10.1016/j.jaci.2019.11.011 31910983 PMC6986782

[13] Warners MJ Hindryckx P Levesque BG Parker CE Shackelton LM Khanna R Systematic review: disease activity indices in eosinophilic esophagitis. Am J Gastroenterol. (2017) 112(11):1658–69. 10.1038/ajg.2017.363 29039850

[14] Wechsler JB Bolton SM Amsden K Wershil BK Hirano I Kagalwalla AF. Eosinophilic esophagitis reference score accurately identifies disease activity and treatment effects in children. Clin Gastroenterol Hepatol. (2018) 16(7):1056–63. 10.1016/j.cgh.2017.12.019 29248734 PMC6003847

[15] Bolton SM Kagalwalla AF Wechsler JB. Eosinophilic esophagitis in children: endoscopic findings at diagnosis and post-intervention. Curr Gastroenterol Rep. (2018) 20(1):1–7. 10.1007/s11894-018-0607-z 29492720 PMC6448395

[16] Dellon ES Cotton CC Gebhart JH Higgins LL Beitia R Woosley JT Accuracy of the eosinophilic esophagitis endoscopic reference score in diagnosis and determining response to treatment. J R Stat Soc Series B Stat Methodol J R STAT SOC B. (2017) 14(1):31–9. 10.1016/j.cgh.2015.08.040.Accuracy PMC4690779 26404868

[17] Benjamini Y Hochberg Y. Controlling the false discovery rate: a practical and powerful approach to multiple testing. J R Stat Soc Ser B. (1995) 57(1):289–300. 10.1111/j.2517-6161.1995.tb02031.x

[18] R Core Team. R: A language and environment for statistical computing. (2020). https://www.r-project.org/

[19] Ancellin M Ricolfi-Waligova L Clerc-Urmès I Schweitzer C Maudinas R Bonneton M Management of eosinophilic esophagitis in children according to atopic status: a retrospective cohort in northeast of France. Arch Pediatr. (2020) 27(3):122–7. 10.1016/j.arcped.2020.02.001 32192814

[20] Hamilton JD Harel S Swanson BN Brian W Chen Z Rice MS Dupilumab suppresses type 2 inflammatory biomarkers across multiple atopic, allergic diseases. Clin Exp Allergy. (2021) 51(7):915–31. 10.1111/cea.13954 34037993 PMC8362102

[21] Hirano I Dellon ES Hamilton JD Collins MH Peterson K Chehade M Efficacy of dupilumab in a phase 2 randomized trial of adults with active eosinophilic esophagitis. Gastroenterology. (2020) 158(1):111–122.e10. 10.1053/j.gastro.2019.09.042 31593702

[22] Drislane C Irvine AD. The role of filaggrin in atopic dermatitis and allergic disease. Ann Allergy Asthma Immunol. (2020) 124(1):36–43. 10.1016/j.anai.2019.10.008 31622670

[23] Franciosi JP Mougey EB Dellon ES Gutierrez-Junquera C Fernandez-Fernandez S Venkatesh RD Proton pump inhibitor therapy for eosinophilic esophagitis: history, mechanisms, efficacy, and future directions. J Asthma Allergy. (2022) 15:281–302. 10.2147/JAA.S274524 35250281 PMC8892718

[24] Capucilli P Hill DA. Allergic comorbidity in eosinophilic esophagitis: mechanistic relevance and clinical implications. Clin Rev Allergy Immunol. (2019) 57(1):111–27. 10.1007/s12016-019-08733-0 30903437 PMC6626558

[25] Letner D Farris A Khalili H Garber J. Pollen-food allergy syndrome is a common allergic comorbidity in adults with eosinophilic esophagitis. Dis Esophagus. (2018) 31(2):1–8. 10.1093/dote/dox122 29087472

[26] Mahdavinia M Bishehsari F Hayat W Elhassan A Tobin MC Ditto AM. Association of eosinophilic esophagitis and food pollen allergy syndrome. Ann Allergy Asthma Immunol. (2017) 118(1):116–7. 10.1016/j.anai.2016.10.012 27856094

[27] Gutiérrez-Junquera C Fernández-Fernández S Cilleruelo ML Rayo A Román E. The role of proton pump inhibitors in the management of pediatric eosinophilic esophagitis. Front Pediatr. (2018) 6:1–7. 10.3389/fped.2018.00119 29868522 PMC5951960

[28] Votto M Raffaele A De Filippo M Caimmi S Brunero M Riccipetitoni G Eosinophilic gastrointestinal disorders in children and adolescents: a single-center experience. Dig Liver Dis. (2022) 54(2):214–20. 10.1016/j.dld.2021.06.027 34274254

