# Supplementary figures and images for: Pediatric eosinophilic esophagitis outcomes vary with co-morbid eczema and pollen food syndrome
Source: Front Allergy. 2022 Sep 2;3:981961. doi: 10.3389/falgy.2022.981961 (PMC9478188; doi:10.3389/falgy.2022.981961)

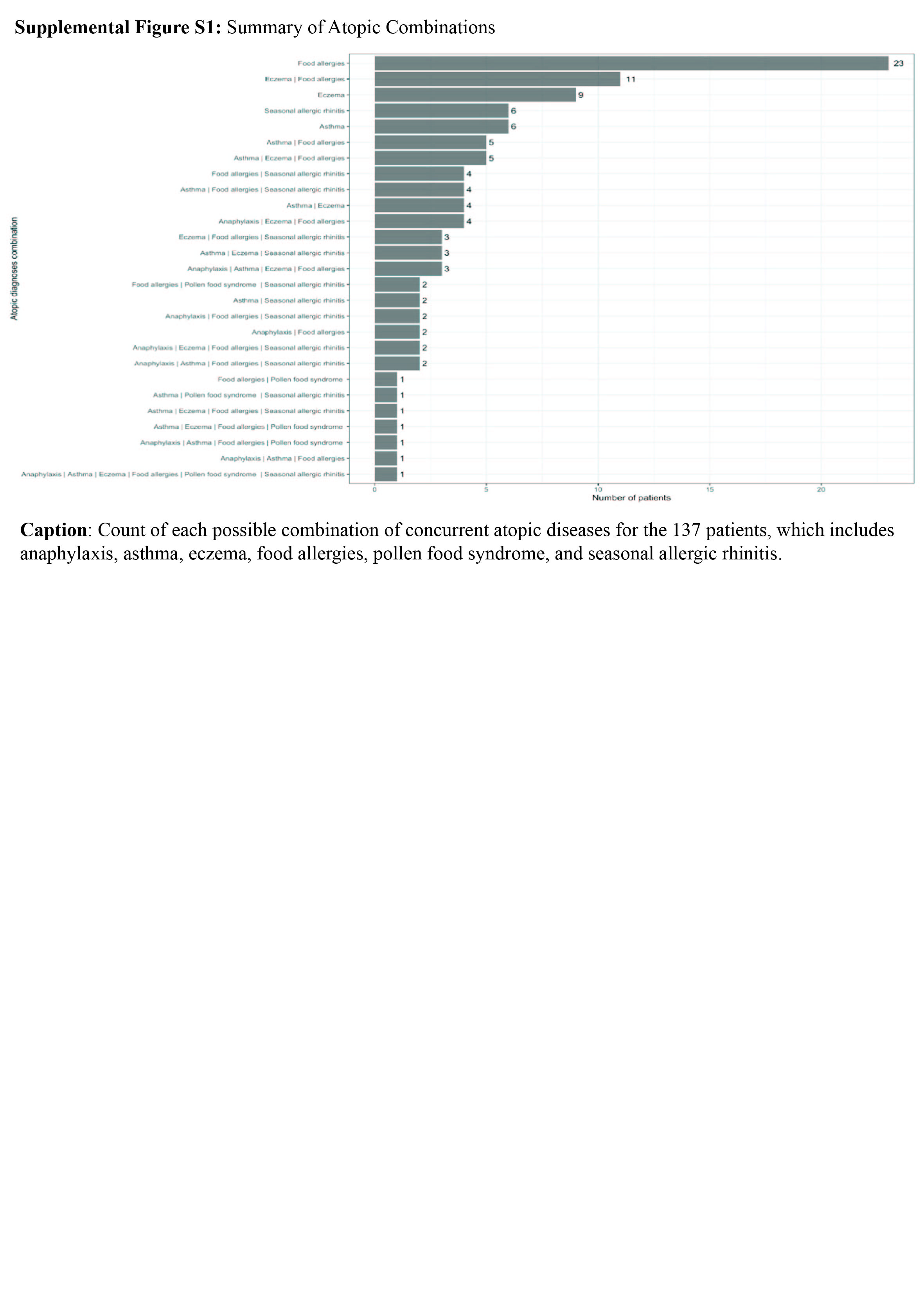

Supplement: Supplementary file 1 [file Image_1_v1.jpeg]

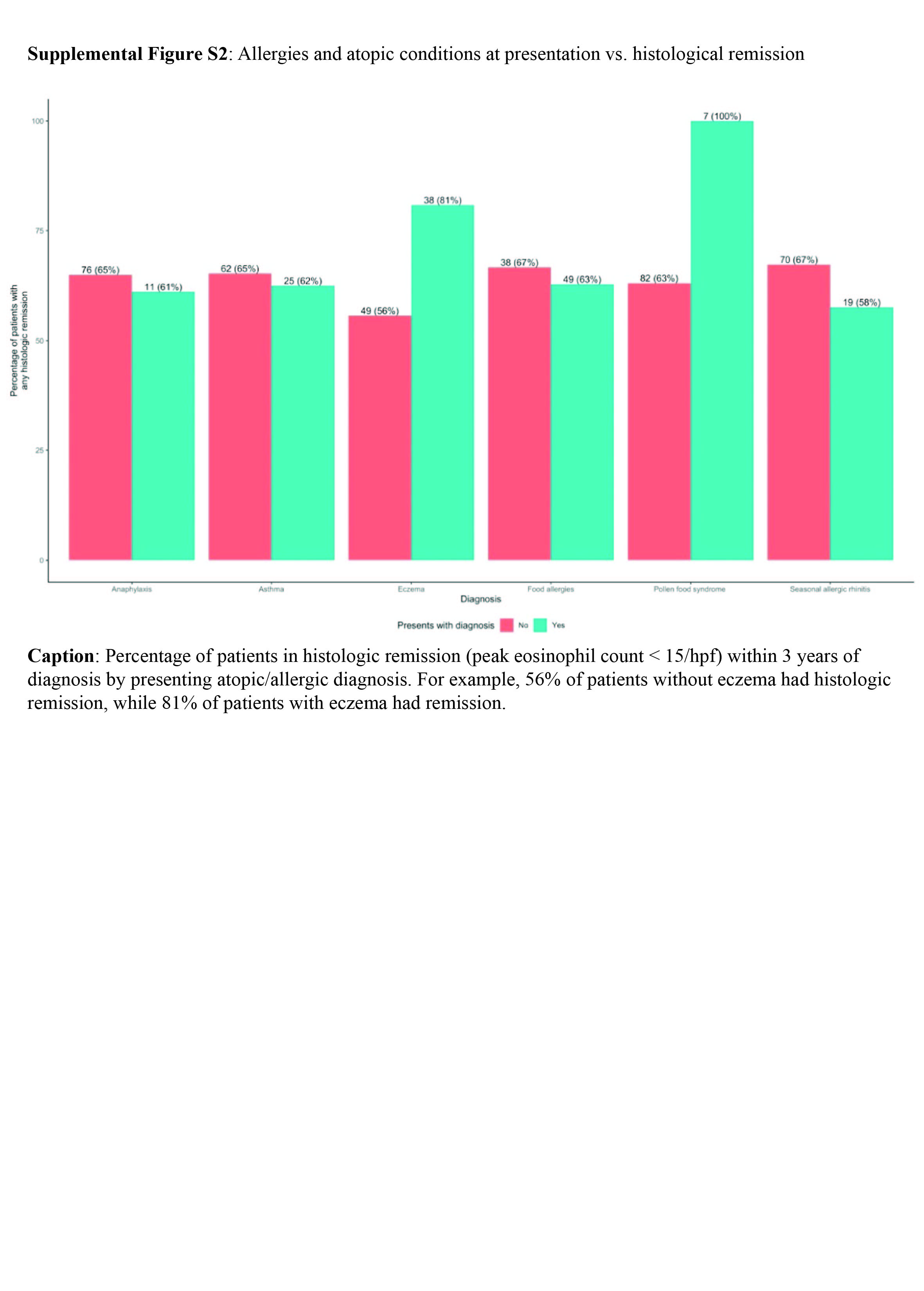

Supplement: Supplementary file 2 [file Image_2_v1.jpeg]
