# Supplementary material for: Pediatric eosinophilic esophagitis outcomes vary with co-morbid eczema and pollen food syndrome
Source: Front Allergy. 2022 Sep 2;3:981961. doi: 10.3389/falgy.2022.981961 (PMC9478188; doi:10.3389/falgy.2022.981961)
Supplement: Supplementary file 3 [file Table_1_v1.docx]

**Supplemental Table S1:**

| **Number of allergic Disorders** | **Remission rate** %(n) | **Average time to remission** (months) (SD) |
| --- | --- | --- |
| 0 (28) | 71% (20) | 12.7 (11) |
| 1 (44) | 52% (23) | 14.2 (11.5) |
| 2 (29) | 76% (22) | 9.99 (8.26) |
| 3 (25) | 64% (16) | 15.7 (13.0) |
| 4 (10) | 70% (7) | 7.46 (7.18) |
| 5 (0) | - | - |
| 6 (1) | 100% (1) | 2.9 |

**Caption:** Remission rate and time to remission (months) by number of concurrent allergic disorders (0-6). Allergic disorders include anaphylaxis, asthma, eczema, food allergies, pollen food syndrome, and seasonal allergic rhinitis.
