# Supplementary material for: Pediatric eosinophilic esophagitis outcomes vary with co-morbid eczema and pollen food syndrome
Source: Front Allergy. 2022 Sep 2;3:981961. doi: 10.3389/falgy.2022.981961 (PMC9478188; doi:10.3389/falgy.2022.981961)
Supplement: Supplementary file 4 [file Table_2_v1.docx]

**Supplemental Table S2:** Demographics of patients with eczema and Pollen Food Syndrome (PFS)

| **Baseline characteristics** | **Overall** | **Eczema** | **Non-Eczema (N=88)** | **p-value** | **PFS** | **Non-PFS (N=130)** | **p-value** |
| --- | --- | --- | --- | --- | --- | --- | --- |
|  | **(N=137)** | **(N=47)** |  |  | **(N=7)** |  |  |
| **Age at diagnosis** |  |  |  |  |  |  |  |
| Median [Min, Max] | 6.4 | 4.6 | 7.1 | 0.004 | 10.9 | 6.1 | 0.085 |
|  | [0.7, 17.0] | [0.8, 17.0] | [0.7, 16.7] |  | [1.48, 13.8] | [0.7, 17.0] |  |
| **Male** | 102 (74.5%) | 36 (76.6%) | 65 (73.9%) | 0.84 | 4 (57.1%) | 98 (75.4%) | 0.37 |
| **Race/ethnicity** |  |  |  | 0.24 |  |  | 0.17 |
| Asian | 16 (11.7%) | 9 (19.1%) | 7(8.0%) |  | 1 (14.3%) | 15 (11.5%) |  |
| Black/African | 3 (2.2%) | 2 (4.3%) | 1 (1.1%) |  | 0 (0%) | 3 (2.3%) |  |
| Caucasian | 45 (32.8%) | 14 (29.8%) | 30 (34.1%) |  | 4 (57.1%) | 41 (31.5%) |  |
| Hispanic/Latino | 8 (5.8%) | 1 (2.1%) | 7 (8.0%) |  | 0 (0%) | 8 (6.2%) |  |
| Multiple | 17 (12.4%) | 6 (12.8%) | 10 (11.4%) |  | 2 (28.6%) | 15 (11.5%) |  |
| Unknown | 48 (35.0%) | 15 (31.9%) | 33 (37.5%) |  | 0 (0%) | 48 (36.9%) |  |
| **BMI (>2yo) or weight-for-length (<2yo) percentiles** | |  |  | 0.19 |  |  | 0.32 |
| <1 | 7 (5.1%) | 5 (10.6%) | 2 (2.3%) |  | 0 (0%) | 7 (5.4%) |  |
| 1-10 | 25 (18.2%) | 11 (23.4%) | 14 (15.9%) |  | 4 (57.1%) | 21 (16.2%) |  |
| 10-25 | 16 (11.7%) | 4 (8.5%) | 12 (13.6%) |  | 0 (0%) | 16 (12.3%) |  |
| 25-75 | 38 (27.7%) | 12 (25.5%) | 26 (29.5%) |  | 2 (28.6%) | 36 (27.7%) |  |
| 75-90 | 12 (8.8%) | 6 (12.8%) | 6 (6.8%) |  | 0 (0%) | 12 (9.2%) |  |
| 90+ | 11 (8.0%) | 2 (4.3%) | 8 (9.1%) |  | 0 (0%) | 11 (8.5%) |  |
| **Atopic symptoms** |  |  |  |  |  |  |  |
| Anaphylaxis | 18 (13.1%) | 10 (21.3%) | 8 (9.1%) | 0.06 | 2 (28.6%) | 16 (12.3%) | 0.24 |
| Asthma | 40 (29.2%) | 18 (38.3%) | 22 (25.0%) | 0.12 | 4 (57.1%) | 36 (27.7%) | 0.2 |
| Eczema | 47 (34.3%) | 47 (100%) | 0 (0%) | - | 2 (28.6%) | 45 (34.6%) | 0.99 |
| Seasonal allergic rhinitis | 33 (24.1%) | 10 (21.3%) | 23 (26.1%) | 0.68 | 4 (57.1%) | 29 (22.3%) | 0.06 |
| Food allergies | 78 (56.9%) | 31 (66.0%) | 47 (53.4%) | 0.2 | 6 (85.7%) | 72 (55.4%) | 0.24 |
| Pollen Food Syndrome | 7 (5.1%) | 2 (4.3%) | 5 (5.7%) | 0.99 | 7 (100%) | 0 | - |
| Unknown allergies | 5 (3.6%) | 1 (2.1%) | 4 (4.5%) | 0.66 | 1 (14.3%) | 4 (3.1%) | 0.23 |
| **Diagnosis of, n (%)** |  |  |  |  |  |  |  |
| Celiac disease | 1 (0.7%) | 0 (0%) | 1 (1.1%) | - | 0 (0%) | 1 (1.1%) | - |
| EGID (eosinophilic gastro-intestinal disorder | 9 (6.6%) | 5 (10.6%) | 3 (3.4%) | 0.15 | 0 (0%) | 9 (6.9%) | - |
| Esophageal malformation (esophageal atresia or trachea-esophageal fistula), n (%) | 6 (4.4%) | 1 (2.1%) | 4 (4.5%) | 0.66 | 0 (0%) | 6 (4.6%) | - |
| **Family history of, n (%)** |  |  |  |  |  |  |  |
| EoE | 6 (4.4%) | 2 (4.3%) | 4 (4.5%) | 0.99 | 1 (14.3%) | 5 (3.8%) | 0.27 |
| Atopic condition | 56 (40.9%) | 19 (40.4%) | 36 (40.9%) | 0.99 | 5 (71.4%) | 51 (39.2%) | 0.32 |
| **Peak eosinophil count on endoscopy** | |  |  | 0.39 |  |  | 0.002 |
| Median [range] | 48 | 55 | 45 |  | 35 | 49.5 |  |
|  | [14, 216] | [14, 200] | [15, 216] |  | [25, 50] | [14, 216] |  |
| **Acute presentation in ED/IP admission** | |  |  | 0.55 |  |  | - |
| n (%) | 15 (10.9%) | 6 (12.8%) | 8 (9.1%) |  | 0 (0%) | 15 (11.5%) |  |
| **EGD gross findings** |  |  |  |  |  |  |  |
| Stricture/Narrowing | 8 (5.8%) | 1 (2.1%) | 7 (8.0%) | 0.26 | 0 | 8 (6.2%) | - |
| Rings/trachealization | 18 (13.1%) | 5 (10.6%) | 13 (14.8%) | 0.79 | 2 (28.6%) | 16 (12.3%) | 0.14 |
| Linear furrow | 66 (48.2%) | 18 (38.3%) | 47 (53.4%) | 0.13 | 3 (42.9%) | 63 (48.5%) | 0.99 |
| Mucosal fragility | 14 (10.2%) | 5 (10.6%) | 9 (10.2%) | 0.99 | 0 | 14 (10.8%) | - |
| Exudate/Microabscess | 38 (27.7%) | 9 (19.1%) | 28 (31.8%) | 0.19 | 2 (28.6%) | 36 (27.7%) | 0.62 |
| Food impaction | 6 (4.4%) | 1 (2.1%) | 5 (5.7%) | 0.66 | 0 | 6 (4.6%) | - |
| Erythema | 10 (7.3%) | 5 (10.6%) | 5 (5.7%) | 0.3 | 0 | 10 (7.7%) | - |
| Edema | 13 (9.5%) | 2 (4.3%) | 11 (12.5%) | 0.21 | 1 (14.3%) | 11 (12.5%) | 0.21 |
| **Total number of pediatric GI endoscopies** | 3 [1,7] | 3 [2,7] | 3 [1,7] | 0.11 | 3 [2,4] | 3 [1,7] | 0.41 |

**Caption:** P-values represent the difference between groups using a standard two-sample t-test for continuous variables and fisher’s exact test for categorical variables. Statistical testing was done for eczema vs. the rest of the cohort and Pollen Food Syndrome (PFS) vs. the rest of the cohort.
