# Supplementary material for: Pediatric eosinophilic esophagitis outcomes vary with co-morbid eczema and pollen food syndrome
Source: Front Allergy. 2022 Sep 2;3:981961. doi: 10.3389/falgy.2022.981961 (PMC9478188; doi:10.3389/falgy.2022.981961)
Supplement: Supplementary file 5 [file Table_3_v1.docx]

**Supplemental Table S3:** Demographics of patients with Pollen Food Syndrome (PFS)

| **PFS Patient #** | **Months to remission** | **Anaphylaxis** | **Asthma** | **Eczema** | **Seasonal allergic rhinitis** | **Food allergies** | **Unknown allergies** | **Remission treatment** |
| --- | --- | --- | --- | --- | --- | --- | --- | --- |
| 1 | 5.7 | No | Yes | Yes | No | Yes | No | Dietary + PPI |
| 2 | 3 | Yes | Yes | No | No | Yes | Yes | No Treatment |
| 3 | 13.1 | No | No | No | Yes | Yes | No | Dietary + Topical |
| 4 | 15 | No | No | No | No | Yes | No | Dietary |
| 5 | 2.9 | Yes | Yes | Yes | Yes | Yes | No | Dietary + PPI + Topical |
| 6 | 3.8 | No | No | No | Yes | Yes | No | Dietary + Topical |
| 7 | 2.1 | No | Yes | No | Yes | No | No | PPI + Topical |

**Caption:** Co-diagnoses and treatment that induced remission for the 7 patients with PFS.
