# Supplementary material for: Pediatric eosinophilic esophagitis outcomes vary with co-morbid eczema and pollen food syndrome
Source: Front Allergy. 2022 Sep 2;3:981961. doi: 10.3389/falgy.2022.981961 (PMC9478188; doi:10.3389/falgy.2022.981961)
Supplement: Supplementary file 6 [file Table_4_v1.docx]

**Supplemental Table S4:** Demographics of PPI responsive vs. PPI non-responsive patients

| **Baseline characteristics** | **Overall** | **PPI** | **PPI Not-** | **p-value** |
| --- | --- | --- | --- | --- |
|  | **(N=55)** | **Responsive** | **Responsive** |  |
|  |  | **(N=41)** | **(N=14)** |  |
| **Age at diagnosis** |  |  |  | 0.98 |
| Mean (SD) | 6.48 (4.34) | 6.49 (4.44) | 6.45 (4.21) |  |
| **Male** | 39 (70.9%) | 30 (73.2%) | 9 (64.3%) | 0.52 |
| **Race/ethnicity** |  |  |  | 0.056 |
| Asian | 7 (12.7%) | 5 (12.2%) | 2 (14.3%) |  |
| Black/African | 1 (1.8%) | 1 (2.4%) | 0 (0%) |  |
| Caucasian | 15 (27.3%) | 13 (31.7%) | 2 (14.3%) |  |
| Hispanic/Latino | 3 (5.5%) | 0 (0%) | 3 (21.4%) |  |
| Multiple | 9 (16.4%) | 8 (19.5%) | 1 (7.1%) |  |
| Unknown | 20 (36.4%) | 14 (34.1%) | 6 (42.9%) |  |
| **BMI (>2yo) or weight-for-length (<2yo) percentiles** | | |  | 0.79 |
| <1 | 2 (3.6%) | 2 (4.9%) | 0 (0%) |  |
| 10 | 11 (20.0%) | 9 (22.0%) | 2 (14.3%) |  |
| 25-Oct | 5 (9.1%) | 3 (7.3%) | 2 (14.3%) |  |
| 25-75 | 15 (27.3%) | 10 (24.4%) | 5 (35.7%) |  |
| 75-90 | 6 (10.9%) | 4 (9.8%) | 2 (14.3%) |  |
| 90+ | 3 (5.5%) | 3 (7.3%) | 0 (0%) |  |
| Missing | 13 (23.6%) | 10 (24.4%) | 3 (21.4%) |  |
| **Atopic symptoms** |  |  |  |  |
| Anaphylaxis | 7 (12.7%) | 5 (12.2%) | 2 (14.3%) | 0.99 |
| Asthma | 16 (29.1%) | 13 (31.7%) | 3 (21.4%) | 0.73 |
| Eczema | 20 (36.4%) | 19 (46.3%) | 1 (7.1%) | 0.01 |
| Seasonal allergic rhinitis | 10 (18.2%) | 8 (19.5%) | 2 (14.3%) | 0.99 |
| Food allergies | 33 (60.0%) | 23 (56.1%) | 10 (71.4%) | 0.36 |
| Unknown allergies | 2 (3.6%) | 2 (4.9%) | 0 (0%) | - |
| Pollen food syndrome | 3 (5.5%) | 3 (7.3%) | 0 (0%) | - |
| **Diagnosis of, n (%)** |  |  |  |  |
| EGID (eosinophilic gastro-intestinal disorder | 3 (5.5%) | 2 (4.9%) | 1 (7.1%) | 0.5 |
| Esophageal malformation (esophageal atresia or trachea-esophageal fistula) | 4 (7.3%) | 3 (7.3%) | 1 (7.1%) | 0.99 |
| **Family history of, n (%)** |  |  |  |  |
| EoE | 0 (0%) | 0 (0%) | 0 (0%) | - |
| Atopic condition | 20 (36.4%) | 16 (39.0%) | 4 (28.6%) | 0.37 |
| **Peak eosinophil count on endoscopy** | |  |  |  |
| Mean [range] | 48.5 [14, 200] | 45.4 [15, 111] | 57.4 [14, 200] | 0.4 |
| **Acute presentation in ED/IP admission** | 5 (9.1%) | 3 (7.3%) | 2 (14.3%) | 0.6 |
| **EGD gross findings** |  |  |  |  |
| Stricture/Narrowing | 4 (7.3%) | 3 (7.3%) | 1 (7.1%) | 0.99 |
| Rings/trachealization | 3 (5.5%) | 2 (4.9%) | 1 (7.1%) | 0.56 |
| Linear furrow | 29 (52.7%) | 23 (56.1%) | 6 (42.9%) | 0.7 |
| Mucosal fragility | 6 (10.9%) | 5 (12.2%) | 1 (7.1%) | 0.99 |
| Exudate/Microabscess | 11 (20.0%) | 9 (22.0%) | 2 (14.3%) | 0.99 |
| Food impaction | 2 (3.6%) | 0 (0%) | 2 (14.3%) | - |
| Erythema | 6 (10.9%) | 6 (14.6%) | 0 (0%) | - |
| Edema | 4 (7.3%) | 3 (7.3%) | 1 (7.1%) | 0.99 |
| **Total number of pediatric GI endoscopies** | |  |  | 0.52 |
| Median [Min, Max] | 3 [1, 7] | 2.5 [1, 7] | 3 [2, 7] |  |

**Caption:** P-values represent the difference between groups using a standard two-sample t-test for continuous variables and fisher’s exact test for categorical variables.
